# Late sodium current and calcium homeostasis in arrhythmogenesis

**DOI:** 10.1080/19336950.2020.1854986

**Published:** 2020-12-21

**Authors:** Kornél Kistamás, Tamás Hézső, Balázs Horváth, Péter P Nánási

**Affiliations:** a Department of Physiology, Faculty of Medicine, University of Debrecen, Debrecen, Hungary; b Department of Dental Physiology, Faculty of Dentistry, University of Debrecen, Debrecen, Hungary

**Keywords:** Voltage-gated sodium channels, sodium current, late sodium current, sodium homeostasis, calcium signaling, calcium homeostasis, cardiac arrhythmias, early afterdepolarization, delayed afterdepolarization

## Abstract

The cardiac late sodium current (I_Na,late_) is the small sustained component of the sodium current active during the plateau phase of the action potential. Several studies demonstrated that augmentation of the current can lead to cardiac arrhythmias; therefore, I_Na,late_ is considered as a promising antiarrhythmic target. Fundamentally, enlarged I_Na,late_ increases Na^+^ influx into the cell, which, in turn, is converted to elevated intracellular Ca^2+^ concentration through the Na^+^/Ca^2+^ exchanger. The excessive Ca^2+^ load is known to be proarrhythmic. This review describes the behavior of the voltage-gated Na^+^ channels generating I_Na,late_ in health and disease and aims to discuss the physiology and pathophysiology of Na^+^ and Ca^2+^ homeostasis in context with the enhanced I_Na,late_ demonstrating also the currently accessible antiarrhythmic choices.

## Introduction

The cardiac action potential (AP) is composed of several ion currents. During the initial depolarization, voltage-gated Na^+^ channels open to further depolarize the membrane. This is followed by a tightly regulated process, in which L-type Ca^2+^ channels open to let the Ca^2+^ ions flow into the cell (L-type Ca^2+^ current, I_Ca,L_), so that the contraction can occur in the process called Ca^2+^-induced Ca^2+^ release (CICR) by opening the ryanodine receptors (RyR). Besides the depolarizing inward currents, a number of outwardly driven K^+^ currents repolarize the membrane. During repolarization, Ca^2+^ is removed from the cytoplasm; therefore, complete relaxation of the cell occurs while the membrane potential returns to its resting value so that the next AP can be elicited [1–3].

**Figure 1.**
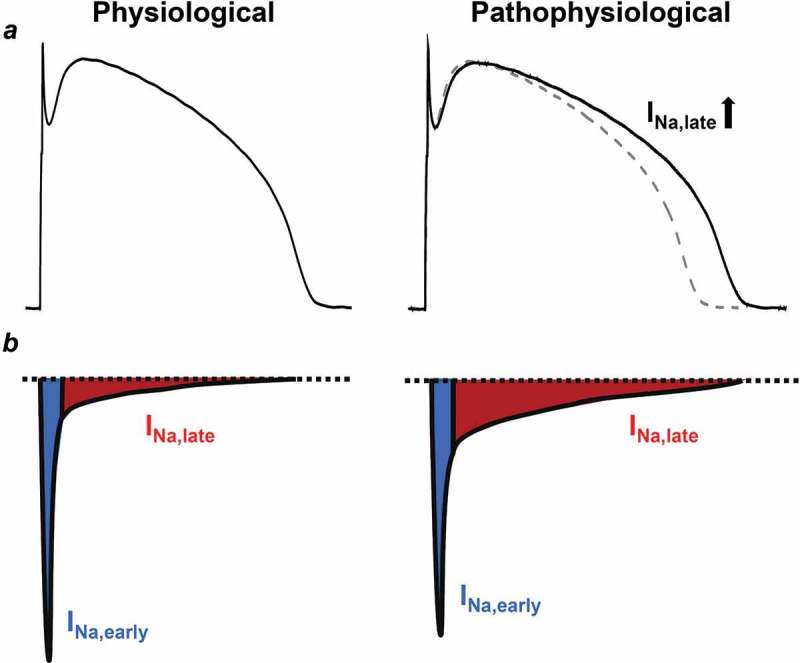
Comparison of the physiological and the pathological I_Na,late_ in ventricular cardiomyocytes. (a) Ventricular action potentials recorded from healthy and diseased hearts. Diseased I_Na,late_ is increased causing a longer action potential. Dashed line shows the control action potential. (b) Representative electrophysiological recordings of the I_Na_ in normal and diseased myocytes. Blue shows the early, peak component of the I_Na_ (I_Na,early_), while red shows the sustained, late component of the current (I_Na,late_)

The behavior of Na^+^ current is not monotonic in time. Once the membrane potential reaches the threshold level for the voltage-gated Na^+^ channels, a significant Na^+^ influx depolarizes the membrane and creates the upstroke of the AP. However, this fast, early peak Na^+^ current (I_Na,early_) is rapidly inactivated causing the fast decay of the I_Na,early_ [4,5]. Under certain conditions, Na^+^ channels might recover from inactivation and reopen during the plateau phase of the AP, bringing a further depolarizing Na^+^ influx, termed as the late Na^+^ current (I_Na,late_) (Figure 1) [6]. As I_Na,early_ increases the intracellular Na^+^ concentration [Na^+^]_i_ at the upstroke of the AP, the Na^+^/Ca^2+^ exchange (NCX) switches to its reverse mode and removes Na^+^ from the cell at the cost of intracellular Ca^2+^ load. This reverse mode persists for only a very short period of time and NCX works in its forward mode at the rest of the AP, underlying the vast majority of sarcolemmal Ca^2+^ extrusion [1,7]. I_Na,late_ is a minute, but persistent inward current which is much smaller in amplitude than I_Na,early_ in healthy myocytes. However, under certain pathophysiological conditions, I_Na,late_ can become much larger and might cause Na^+^ (and Ca^2+^) overload leading to arrhythmogenesis (Figure 1). In the present review we aim to discuss the arrhythmogenic role of I_Na,late_ in context with the intracellular Na^+^ and Ca^2+^ overload.

## Voltage-gated sodium channels

**Figure 2.**
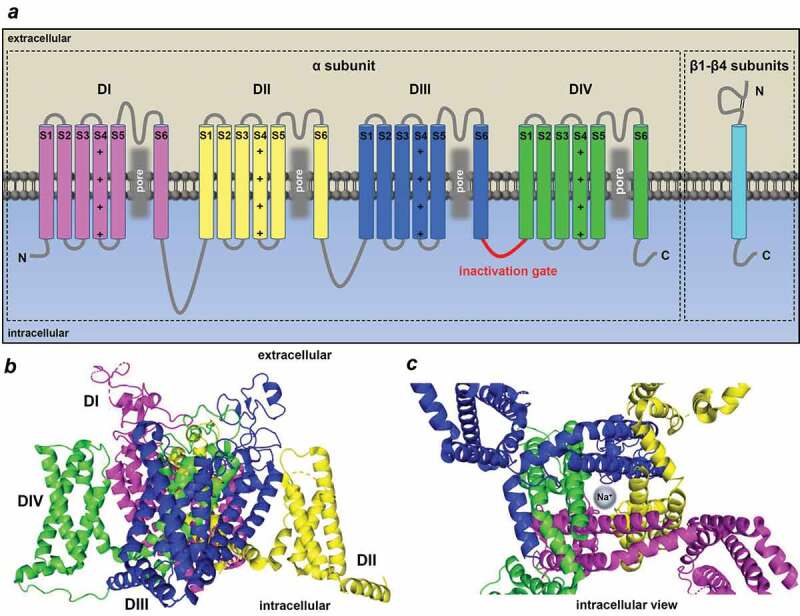
Structure of the cardiac Na_v_1.5 α subunit. (a) Alpha and beta subunits of the cardiac voltage-gated Na^+^ channel isoform showing the four domains of the alpha subunit (DI-DIV) and the six transmembrane segments (S1-S6) in each domain and the auxiliary beta subunits. Grey zone shows the pore-forming domain. Red shows the inactivation gate between the DIII and DIV (IDIII/IV). (b) Side and (c) top (intracellular) view of the cryo-EM structure of the rat Na_v_1.5 α subunit displaying the domains in different colors (PDB ID: 6UZ3) and the channel pore (c) generated by PyMol Software.[182]

Since the original observations by Hodgkin and Huxley on squid giant axon, voltage-gated Na^+^ channels are known to be regulated by changes in the actual membrane potential [8]. If the membrane voltage is favorable for channel opening, the movement of ions is determined by the electrochemical gradient of the ion. Voltage-gated Na^+^ channels consist of a large pore-forming pseudotetrameric α subunit, accessory β subunits and scaffolding proteins (Figure 2(a)). To date there are 9 different α subunits (Na_v_1.1, Na_v_1.2, Na_v_1.3, Na_v_1.4, Na_v_1.5, Na_v_1.6, Na_v_1.7, Na_v_1.8, Na_v_1.9) encoded by 9 different genes (SCN1A, SCN2A, SCN3A, SCN4A, SCN5A, SCN8A, SCN9A, SCN10A, SCN11A) of which the Na_v_1.5 is considered to be the dominant cardiac subtype [9,10]. Na_v_1.5 is relatively insensitive to the Na^+^ channel blocker neurotoxin, tetrodotoxin (TTX) [9,11–13]. However, other TTX sensitive subtypes – such as Na_v_1.1, Na_v_1.2, Na_v_1.3, Na_v_1.4 and Na_v_1.6 – are also reported to be expressed in the heart [14–18]. An α subunit encoded by a specific gene determines not only the channel subtype itself, but also the receptor population of the particular channel. The 6 possible β subunits (β1, β1A, β1B, β2, β3, β4) are encoded by four genes (SCN1B, SCN2B, SCN3B, SCN4B) [19–22]. The α subunit alone is sufficient to form a functional channel; however, the auxiliary β subunits are required for regular channel kinetics and cell surface expression [20]. In fact, as the β subunits modulate the number of the available channels on the cell surface, they play a role in regulating peak current density [20]. Furthermore, β subunits control activation, inactivation, and recovery from inactivation by altering their voltage range [23,24].

Voltage-gated Na^+^ channel α subunits consist of approximately 2000 amino acid residues, creating four domains (DI-DII-DIII-DIV) (Figure 2(a-b)). Each domain is formed by six transmembrane segments (S1-S6). Segments 1–4 (S1-S4) function as the voltage sensor domain of the channel. This domain senses membrane depolarization leading to channel activation. The S1-S4 connects to the channel’s pore-forming domain S5-S6 *via* an intracellular linker. This structure encompasses the central aqueous pore domain (Figure 2(c)). The selectivity filter is also located in the pore domain, recognizing the charge and radius of the ion.

At membrane potentials negative to the threshold of the Na^+^ channel the channel’s open probability is low. Upon depolarization, however, the α subunit undergoes a conformational change, the voltage sensor activates, the activation gate – and therefore the Na^+^ channel – quickly opens, thereby conducting Na^+^ current and resulting in the upstroke of the cardiac AP. A few milliseconds later the channel inactivates quickly as the inactivation gate closes into the channel’s pore domain yielding a nonconducting state [25]. The homologous domains are connected by intracellular interdomain loops (IDI/II, IDII/III and IDIII/IV). Inactivation gate incorporates the smallest interdomain cytoplasmic loop (IDIII/IV) and functions as a lid that locks the pore during inactivation (Figure 2(a)) [25–29]. Normal inactivation is needed to prevent excess depolarization and to ensure timely repolarization. It has also been proposed that the inactivation gate is formed and stabilized as a molecular complex, formed by the IDIII/IV and the C-terminal loop of the α subunit [30]. After depolarization and inactivation, during the repolarization phase, Na^+^ channels recover from inactivation ready to be activated again [31].

## Late sodium current

I_Na,late_ is normally a small but persistent current (Figure 1). It is active during the plateau phase of the cardiac AP; therefore, the current can play a significant role in determining the duration and the shape of the AP (Figure 1(b)) [32–34]. A possible explanation of this discrepancy (i.e., a tiny current causing large effects on the AP) is given by the role of net membrane current. During the plateau phase of the AP the impedance of the cell membrane is high [35] and according to Ohm’s law, at this stage, small changes in net membrane current lead to relatively large changes in the membrane potential, and consequently, in AP duration (APD) [36,37].

**Figure 3.**
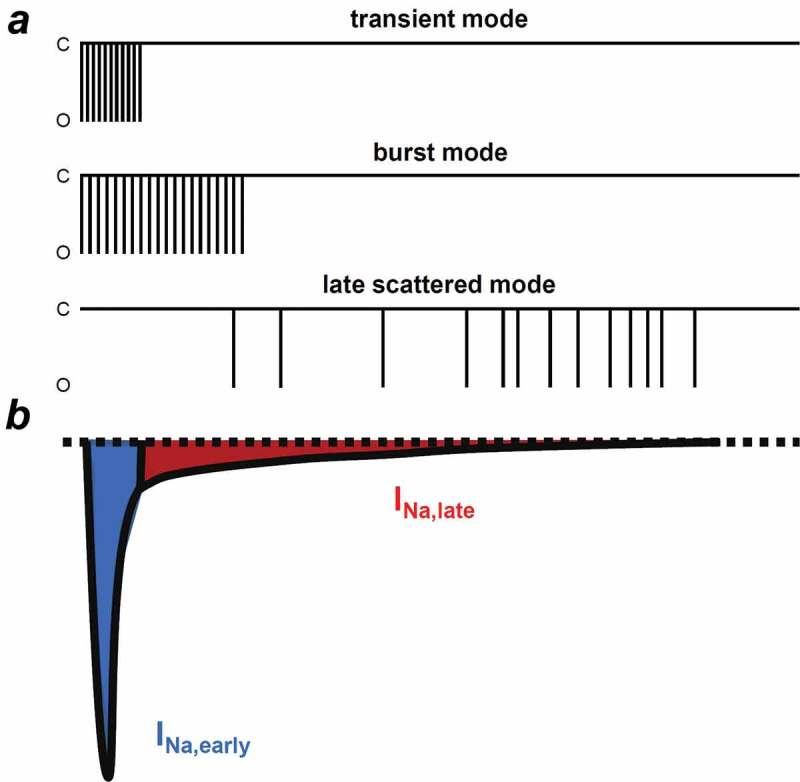
Different gating modes determining the I_Na,early_ and I_Na,late_. (a) Schematic illustrations of the three Na_v_1.5 gating modes. I_Na,early_ is determined by the transient mode. Burst mode and late scattered mode are responsible for I_Na,late_, however, the late scattered mode is the main gating for I_Na,late_. (b) Representative electrophysiological recording of the I_Na_. Blue shows the early, peak component of the I_Na_ (I_Na,early_), while red shows the sustained, late component of the current (I_Na,late_). C, closed and O, opened

Three different gating modes of Na_v_1.5 have been described in ventricular cells (Figure 3) [38]. The transient mode is the main gating mode for the I_Na,early_. Burst mode and late scattered mode are responsible for I_Na,late_; however, burst mode openings decline quickly, leaving the late scattered mode to be the main gating for I_Na,late_ during the plateau phase. Furthermore, several inactivation processes have been proposed, each governing APD, Na^+^ channel steady-state inactivation and Na^+^ flux balance of the cell [39,40]. Fast inactivation takes place only in the first milliseconds and the channel recovers rapidly at negative membrane potentials. This is followed by the intermediate inactivation which recovers slowly compared to the fast inactivation. Slow inactivation from the open state occurs over hundreds of milliseconds and finally, ultraslow inactivation can take seconds.

The window Na^+^ current is a well-known phenomenon characterizing Na^+^ channels. Due to an overlap between steady-state activation and inactivation curves (“window of potentials”), a fraction of Na^+^ channels can recover from inactivation and might reopen. However, considering the voltage range of this window current (approximately – 70 mV), it is far below the physiological plateau potential of the AP, so it is unlikely to play a major role in the I_Na,late_ [34,41–43].

There are marked interspecies differences in the profiles of the I_Na,late_. One practical difference is the shape and duration of the AP, resulting in distinct I_Na,late_ profiles. Our group has recently demonstrated that I_Na,late_ in human and canine ventricular myocytes is markedly disparate from cells isolated from guinea pig hearts [44] or some other mammals including rabbits and pigs [45–47]. The greatest difference was in the time course and profile of the I_Na,late_. In human and canine cells, the amplitude of the I_Na,late_ monotonically decreases during the time course of the AP. On the contrary, guinea pig cells show a different current profile, namely, the current amplitude increases during the plateau phase to only decline during the terminal repolarization. In addition, Horváth *et al*. and Hegyi *et al*. reported that the density of the I_Na,late_ is comparable to the major repolarizing K^+^ currents in guinea pig and rabbit myocytes [45,47]. Also in rabbits, the atrial density of I_Na,late_ was greater than measured in the ventricles [48].

I_Na,late_ shows reverse rate-dependent properties, that is, the higher the pacing frequency (i.e. heart rate), the lower the density of the current [37,49,50]. However, the early and late components behave differently. The higher [Na^+^]_i_, observed at high frequency, is mainly determined by the early component as I_Na,late_ recovers slowly from inactivation at rapid pacing [51]. Additionally, at higher frequencies, APD is usually shortened, allowing less time for activation of I_Na,late_. Therefore the shorter the APD the smaller the I_Na,late_ and Na^+^ influx. In contrast, during bradycardia APD is longer and shows greater beat-to-beat variability [52]. Therefore, bradycardia, associated with enhanced I_Na,late_, may strongly be proarrhythmic [45,53,54]. As there are marked interspecies differences in heart rate and APD, species having long APs (e.g. human, canine, guinea pig) are expected to manifest larger I_Na,late_ and Na^+^ influx than species with fundamentally shorter APs (e.g. rat, mouse).

## Late sodium current in disease

In normal, healthy myocytes, the amplitude of I_Na,late_ is much smaller, less than 0.1% of the peak I_Na,early_ [55,56]. However, as stated before, the current is persistent, lasting for 100–400 ms; therefore, the inward charge carried by I_Na,late_ is comparable to I_Na,early_ mediated within 1–2 ms [57,58]. Some papers in the literature refer to this as endogenous I_Na,late_ and is thought to be without any arrhythmic properties.

On the other hand, the density of I_Na,late_ can be increased under many pathophysiological conditions, such as heart failure (HF) [53,59], hypertrophic cardiomyopathy, inherited long QT syndrome 3 (LQTS-3) [16,34,53,60,61], oxidative stress, or atrial fibrillation (AF) with intracellular Ca^2+^ handling abnormalities [62]. Moreover, even a low heart rate or pharmacological interventions can elevate I_Na,late_ [63]. I_Na,late_ is also augmented in myocardial ischemia/reperfusion injury [18,62] and in the presence of characteristic components of ischemia (e.g. hypoxia, ischemic metabolites, hydrogen peroxide) as documented in voltage clamp experiments [64–67].

The AP lengthening effect of the augmented I_Na,late_ can also be observed in HF [68]. The increased I_Na,late_ results in a Na^+^ overload, which, in turn, leads to elevation of intracellular Ca^2+^ concentration [Ca^2+^]_i_. The concomitant abnormal conduction can cause sudden death in HF patients. Conduction velocity is determined also by the Na^+^ channel function [69]. In the ventricular conductive system (Purkinje fibers) – in contrast to ventricular myocardium – slow pacing generates a higher, while fast pacing results in a significantly lower I_Na,late._ This transmural inhomogeneity may be a trigger for cardiac arrhythmias [70]. All these mechanisms may lead to complex pathological electrical and mechanical performance, such as contractile dysfunction [71], disturbed myocardial energetics [72] and arrhythmias [73]. Increased I_Na,late_ is most arrhythmogenic in those cases, where the repolarization reserve is already compromised, such as during treatment with I_Kr_ inhibitors [74], or in the remodeled myocardium.

In the above mentioned diseases several pathways can play a role in the alteration of I_Na,late_. I_Na,late_ can be elevated by reactive oxygen species (ROS), H_2_O_2_ [60,66,75], acidosis [76,77], hypoxia [78,79], or nitric oxide (NO) [80]. Furthermore, I_Na,late_ is also altered by transcriptional regulation [81], N-glycosylation [82,83], phosphorylation on tyrosine residues [84] or arginine methylation [85]. Modulation of channel function can also be achieved by mechanosensitivity [86,87], β-adrenergic stimulation [47], or CaMKII [88–90].

## Calcium and sodium homeostasis

In the case of facilitated sarcolemmal Na^+^ entry to the cytoplasm, [Na^+^]_i_ is going to increase with a concomitant rise in the [Ca^2+^]_i_, which is considered to be arrhythmogenic [3]. Furthermore, high Ca^2+^ *via* the Ca^2+^/calmodulin-dependent protein kinase II (CaMKII) – protein kinase C (PKC) pathway can further increase I_Na,late_ thereby initiating a vicious circle, leading to spatial heterogeneity of Ca^2+^ transients and triggered activities [90–92]. Therefore, a better understanding of the effects of the elevated I_Na,late_ on Na^+^ and Ca^2+^ homeostasis is critically important.

The Na^+^ balance of a healthy myocyte consists of influx and efflux of Na^+^. The main sources for Na^+^ influx from the extracellular compartment are the Na^+^ channels, the NCX and the Na^+^/H^+^ exchanger (NHX). Na^+^ leaves the cell *via* the Na^+^/K^+^ pump (NKP) and NCX operating in its reverse mode. Approximately 25% of the Na^+^ entry is produced by the Na^+^ channels, equally distributing between I_Na,early_ and I_Na,late_, while NCX provides about 60% of total Na^+^ influx [93]. Additionally, other routes for Na^+^ fluxes may contribute to a minor extent. The Na^+^ and Ca^2+^ homeostasis are strictly coupled processes [93,94]. Beyond the conversion of the elevated intracellular Na^+^ to Ca^2+^ by the NCX, it is easy to consider that a sustained depolarization above −40 mV, due to the augmented I_Na,late_, may increase the open probability of L-type Ca^2+^ channels. In other words, a longer AP causes higher Ca^2+^ influx and Ca^2+^ load [95–99].

**Figure 4.**
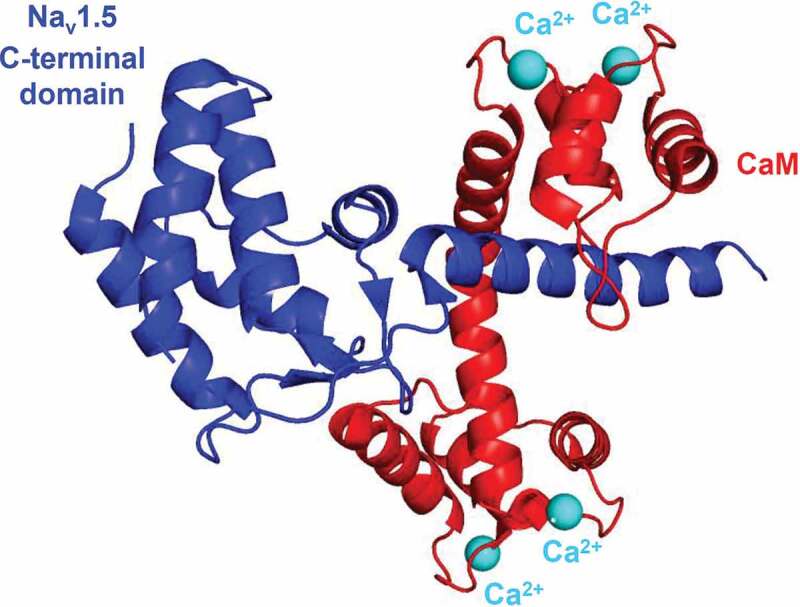
Connection of the Ca^2+^/CaM complex to the Na^+^ channel. Crystal structure shows the human Na_v –_ Ca^2+^/CaM complex (PBD ID: 6MUD [106]) designed by PyMol Software [106]. Blue shows the C-terminal region of the Na_v_1.5 α subunit, while red shows the CaM with 4 Ca^2+^ ions bound (cyan spheres). CaM, calmodulin

Enhancement of the I_Na,late_ can be achieved through the Ca^2+^ – calmodulin (CaM) – CaMKII pathway. CaM and CaMKII can regulate the channel individually and cooperatively as well [90,100,101]. CaM modulates Na^+^ channel function by binding to an IQ domain of the channel protein at the C-terminus and enhances slow inactivation (Figure 4) [102–104]. CaM decreases the sustained I_Na,late_ during depolarization, therefore reduces the risk of arrhythmias [105]. Until recently, understanding of the association of Na^+^ channels and CaM was limited, as most of the studies applied the Ca^2+^-free CaM, apocalmodulin (apoCAM). The binding site for both Ca^2+^-free and Ca^2+^-occupied CaM is the IQ motif [106]. Wang *et al*., however, recently demonstrated that Ca^2+^ induces a conformational switch in the CaM, in which the N-lobe of the CaM contacts with the distal IQ motif of the C-terminal domain of the Na^+^ channel, while the C-lobe of the CaM (Ca^2+^ free) remains anchored to the IQ motif and this action is isoform-specific [107]. There are controversial studies on whether Ca^2+^ alone can regulate Na^+^ channels [102,105,108]. Gardill *et al*. concluded that the position of the EF-hand domain regulates Ca^2+^-dependent inactivation [106]. Anomalous diffraction studies, on the other hand, proposed a Ca^2+^-sensor role for CaM rather than the EF-hand of the Na^+^ channel C-terminal domain [109]. CaMKIIδ – the predominant cardiac isoform – may also alter the inactivation properties of Na^+^ channels. The Ca^2+^-CaM complex activates CaMKII which, in turn, phosphorylates the Na^+^ channel and enhances I_Na,late_ [88–90,110]. CaMKII increases intermediate inactivation and slows recovery, but slows the open state inactivation of I_Na,early_ and increases I_Na,late_, increasing ultimately [Na^+^]_i_ [110–112]. Na^+^ channel regulation by CaMKII can also take place by association with the channel and by phosphorylation of the channel proteins [111]. In rabbits, phosphorylation of Na^+^ channels by endogenous CaMKII occurs even at physiological Ca^2+^ levels [111]. In addition to the effects on Na^+^ channels, phosphorylation by CaMKII enhances protein kinase A (PKA), I_Ca,L_ and sarcoendoplasmic reticulum Ca-ATPase (SERCA) and activates RyR (Figure 4) [113–118]. Sustained depolarization by the augmented I_Na,late_ also contributes to cell Ca^2+^gain. These altogether increase the sarcoplasmic reticulum (SR) Ca^2+^ content and the open probability of RyR, therefore giving substrate for spontaneous Ca^2+^ release events [112,119].

In HF (both human HF and animal model of HF) expression and activity of CaMKII are increased, which may be proarrhythmogenic [120–122]. Furthermore, it has been shown that transgenic overexpression of cytosolic CaMKII can induce HF [117,118]. Acute overexpression of CaMKII enhances I_Na,late_ and increases [Na^+^]_i_, slows inactivation of I_Na,early_ and recovery from inactivation, while shifting steady-state inactivation to more negative membrane potential in a Ca^2+^-dependent manner [111]. All these effects of acute overexpression of CaMKII can be hindered by CaMKII inhibition. In undiseased ventricular cells, it has been shown that a fourfold increase in Na^+^ current density was required to achieve a significant increase in [Na^+^]_i_ [61,123]. Wei *et al*. demonstrated that phosphorylation of CaMKII and the expression of Na_v_1.5 channel protein has been significantly elevated in the left ventricle upon treatment with the Ca^2+^ channel activator Bay K 8644 and the Na^+^ channel activator sea anemone toxin II (ATX-II). These effects were readily reversible by the application of TTX [124]. Bay K 8644 and ATX-II increased the APD more powerfully when applied simultaneously and caused ventricular tachycardia with high incidence. This synergistic connection between high [Ca^2+^]_i_ and high [Na^+^]_i_ potentiates their arrhythmogenic activities.

Mitochondria are important Ca^2+^ buffering stores [125,126]. They contribute to Ca^2+^ homeostasis by taking up cytosolic Ca^2+^ *via* the mitochondrial Ca^2+^ uniporter (MCU) or releasing Ca^2+^ through the mitochondrial NCX (mNCX), the latter being an [Na^+^]_i_ sensitive transporter [67,127,128]. Under conditions of Ca^2+^ overload, as suggested by Ronchi *et al*. in a simulated ischemia protocol in rat ventricular myocytes, blockade of the sarcolemmal NCX turned mitochondria into a Ca^2+^ source from being a Ca^2+^ sink. It was concluded that during Ca^2+^ overload mitochondria may play a role in providing extra cytosolic Ca^2+^ and may be responsible for the I_Na,late_ mediated perturbation of the intracellular milieu [129].

**Figure 5.**
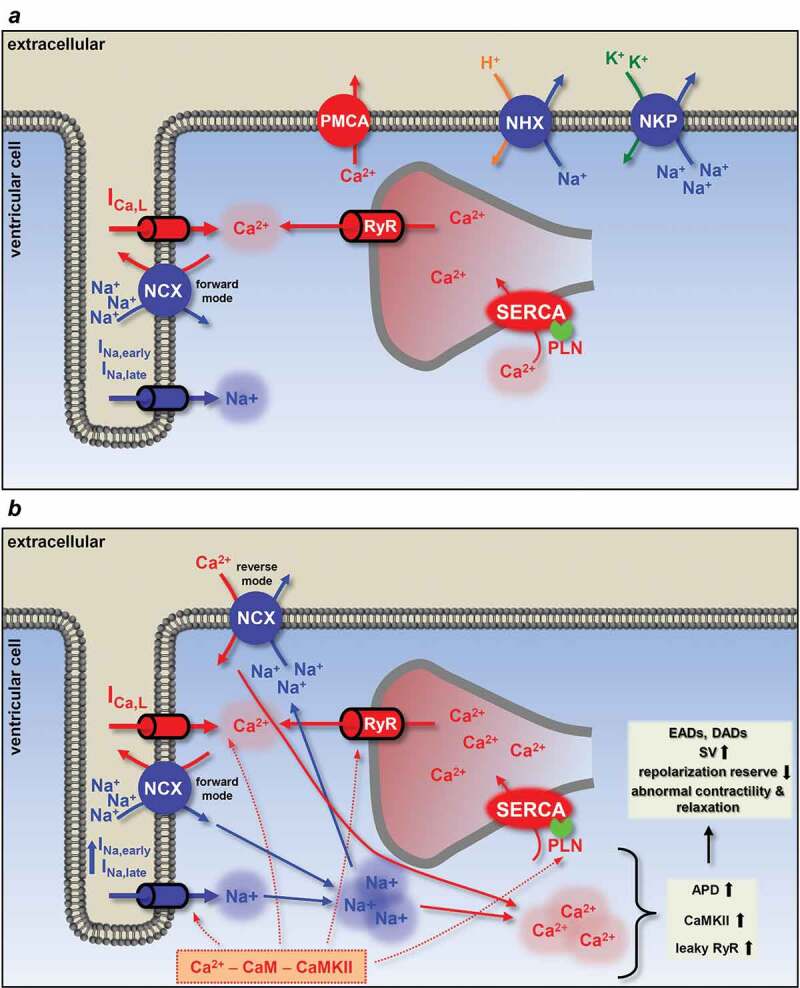
Schematic illustration of the physiological and pathophysiological processes leading to arrhythmias upon increased I_Na,late_. (a) In a healthy myocyte, excitation-contraction coupling controls contraction by periodically increasing and decreasing the intracellular Ca^2+^ concentration. (b) If I_Na,late_ is elevated, as in the case of many diseases, intracellular Na^+^ and a concomitant Ca^2+^ overload may lead to arrhythmias. High intracellular Na^+^ concentration can activate the reverse mode NCX to further load the cell with Ca^2+^. Ca^2+^ overload and the longer action potential duration predispose the cell to proarrhythmic events. Red arrows show Ca^2+^ related, while blue arrows show Na^+^ related processes. Dashed lines indicate the phosphorylation targets of the Ca^2+^ – CaM – CaMKII pathway. APD, action potential duration; CaM, calmodulin; CaMKII, Ca/calmodulin-dependent protein kinase II; DAD, delayed afterdepolarization; EAD, early afterdepolarization; I_Ca,L_, L-type Ca^2+^ current; I_Na,early_, the fast, early component of the Na^+^ current; I_Na,late_, the persistent, late component of the Na^+^ current; NCX, Na^+^/Ca^2+^ exchange; NHX, Na^+^/H^+^ exchanger; NKP, Na^+^/K^+^ pump; PLN, phospholamban; PMCA, plasma membrane Ca^2+^-ATPase; RyR, ryanodine receptor; SERCA, sarcoplasmic reticulum Ca^2+^-ATPase; SV, short term beat-to-beat variability of action potential duration

**Figure 6.**
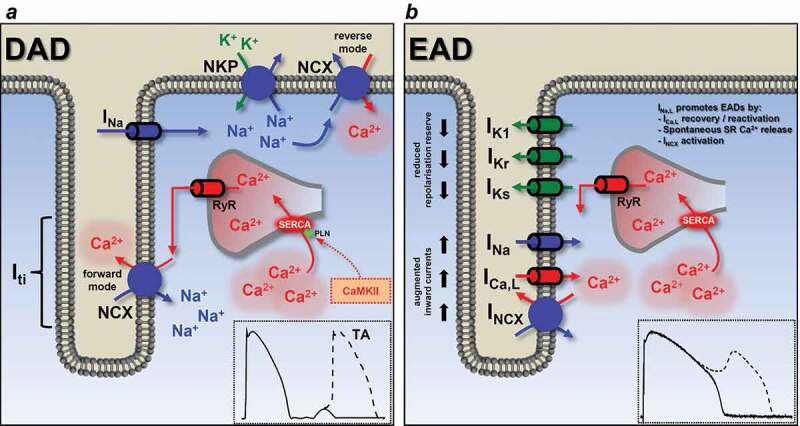
Basic mechanisms for early and delayed afterdepolarizations. (a) Factors involved in the generation of delayed afterdepolarizations (DAD). Increased [Na^+^]_i_ elevates [Ca^2+^]_i_ (and SR Ca^2+^ content) by switching NCX to reverse mode if the functional reserve of the NKP is reached. CaMKII phosphorylates phospholamban, also increasing SR Ca^2+^ content. High SR Ca^2+^ causes spontaneous Ca^2+^ release via the ryanodine receptors. This abnormal Ca^2+^ signaling switches NCX to forward mode, generating the transient inward current and this membrane depolarization can lead to triggered activity. Usually happens at high frequency, during diastole. Membrane potential recording shows a typical DAD. (b) Early afterdepolarization (EAD) occurs when the outward currents are reduced (reduced repolarization reserve) and/or the inward currents are enhanced. I_Na,late_ promotes EAD generation by the reactivation of I_Ca,L_ during the plateau phase, NCX activation and SR Ca^2+^ overload. Membrane potential recording shows a typical phase EAD. EAD, early afterdepolarization; DAD, delayed afterdepolarization; I_Ca,L_, L-type Ca^2+^ current; I_K1_, inward rectifier K^+^ current; I_Kr_, rapid component of delayed rectifier K^+^ current; I_Ks_, slow component of delayed rectifier K^+^ current; I_Na_, Na^+^ current; I_NCX_, Na^+^/Ca^2+^ exchange; I_ti_, transient outward current; NKP, Na^+^/K^+^ pump; RyR, ryanodine receptor; SR, sarcoplasmic reticulum; SERCA, sarcoplasmic reticulum Ca^2+^-ATPase; TA, triggered activity

Elevated [Na^+^]_i_, when exceeding the functional reserve of the NKP, increases [Ca^2+^]_i_ by switching NCX to a reverse mode operation with a consequent loading of the SR Ca^2+^ stores (Figure 5). Even a relatively small, a few millimolar increase in [Na^+^]_i_ slows Ca^2+^ extrusion by NCX [93]. In addition, CaMKII phosphorylates the SERCA regulatory protein phospholamban (PLN), thereby augmenting SERCA activity and further gaining SR Ca^2+^ content [118]. This predisposes SR and RyR to spontaneous Ca^2+^ releases which lead to the development of delayed afterdepolarizations (DAD). DADs occur in diastole after full repolarization and are usually the results of intracellular Ca^2+^ overload and spontaneous SR Ca^2+^ release (Figure 6(a)). The abnormal Ca^2+^ release generates a depolarizing current by activating the forward mode of NCX [130]. The development of DADs has clinical importance as they generate triggered activity which contributes to arrhythmogenesis in certain diseases, such as catecholaminergic polymorphic ventricular tachycardia (CPVT), HF or AF [3].

Besides DADs, early afterdepolarizations (EAD) can be generated in the case of abnormal Na^+^ channel function (Figure 6(b)). APD is lengthened upon slower I_Na,early_ inactivation predisposing the cell to the generation of EADs. There are several subtypes of EADs (phase 2, phase 3 and late phase 3 EAD) but, in general, they occur before the terminal repolarization. In most cases, a longer AP (except for the late phase 3 EAD) allows I_Ca,L_ to recover from inactivation generating a positive feedback loop triggering further APs [3]. It is important to note that activation of CaMKII itself may also contribute to the facilitation and reactivation of I_Ca,L_ [131,132]. It has been shown, however, that both the SR Ca^2+^ load with the concomitant spontaneous Ca^2+^ release and the inward depolarizing current delivered by the NCX and the reactivated I_Na_ are also accountable for the generation of EADs [133–135]. Two possible mechanisms have been proposed to explain the EAD generation by I_Na,late_; SR Ca^2+^ overload and the reactivation of I_Ca,L_ during the plateau phase of the AP [63]. Our experiments in guinea pig myocytes showed that I_Na,late_-induced EADs are mediated by spontaneous SR Ca^2+^ release as the first occurrence of EAD precedes – i.e. occurs at more positive membrane potential – the window current voltage range of I_Ca,L_, therefore making I_Ca,L_ reactivation as a key feature less likely [45].

It has been demonstrated in LQTS-3 patients, that a gain-of-function mutation of SCN5A resulted in an enlargement of I_Na,late_ [136]. Besides increasing I_Na,late_, the mutation also caused elevation of [Na^+^]_i_ and [Ca^2+^]_i_ which was associated with a reduction in the forward mode and an increase in the reverse mode activity of NCX [123,137–139]. Other studies have shown high SR Ca^2+^ content and spontaneous diastolic Ca^2+^ transients in isolated cells from LQTS-2 mutant mice [140,141].

Previously, we established the concept of relative short-term beat-to-beat variability of APD (RSV), which might be a novel approach for predicting arrhythmias [52,142]. In those settings, higher RSV is considered to be more arrhythmogenic by increasing the dispersion of refractoriness. Those experiments showed that higher Na^+^ current causes higher variations in the APD, which is in agreement with the proarrhythmic role of I_Na,late_. In our experiments, Na^+^ current was inhibited by TTX and lidocaine, or alternatively, activated by veratridine. Similar increase in beat-to-beat variability was observed under Ca^2+^ overload conditions [143] and in situations where the repolarization reserve has been compromised [52].

I_Na,late_ can be directly or indirectly regulated by Ca^2+^, CaM, and CaMKII. In general, the higher [Ca^2+^]_i_ shifts the steady-state inactivation curve of the Na^+^ current to more positive voltages and increases the availability of the channels at more positive potentials [45,108,144]. Consequently, the buffering of [Ca^2+^]_i_ should decrease I_Na,late_. Our experiments, however, showed that I_Na,late_ is rather influenced by the shape and voltage profile of the AP than by Ca^2+^ itself [45]. On the other hand, inhibition of CaMKII successfully prevented the catecholamine-induced spontaneous Ca^2+^ waves, DADs and EADs while improving contractile function [145–148]. Unfortunately, targeting CaMKII as an antiarrhythmic option is rather difficult considering its immensely complex signaling network.

It has recently been shown that inhibition of the exchange protein directly activated by cAMP (Epac) can induce EADs [149]. The mechanism involves oxidative activation of CaMKII by an increase in cellular reactive oxygen species, ROS an increase in I_Na,late_ and prolongation of APD. ROS activation of CaMKII phosphorylates RyR and Na_v_1.5, leading to SR Ca^2+^ leak through RyR and enhanced I_Na,late_ [150]. Application of ranolazine prevented the proarrhythmic effects: decreased APD and abolished EADs, i.e. the impaired Epac signaling induced arrhythmias.

For a detailed review about the role of Ca^2+^ in arrhythmogenesis see a recent review of Kistamás *et al* [3].

## Antiarrhythmic drug development

It became clear that elevation of I_Na,late_, [Na^+^]_i_, and [Ca^2+^]_i_ is arrhythmogenic; therefore, an effective antiarrhythmic treatment is necessary under these conditions. An obvious objective is the inhibition of voltage-gated Na^+^ channels. However, the disappointing results of the CAST and SWORD studies clarified that blocking a single specific ion channel alone can lead to unexpected adverse effects. Recently, a need for selective inhibitors that are able to distinguish between I_Na,early_ and I_Na,late_ is rather emerging. The selectivity here is critically important since blocking the early component of the current can lead to a decrease in conduction velocity and might lead to conduction block and reentrant arrhythmias [3,151,152].

A number of inhibitors have been developed to date including ranolazine, eleclazine (GS-6615), lidocaine, GS-458967, GS-462808, and F15845, however, mainly ranolazine was used for excessive experimental and clinical studies (Table 1) [153]. The main issue with most of these inhibitors is that they function in a voltage-dependent manner and exert their I_Na,late_ selective effects mainly at lower than physiological membrane potentials. Coming closer to the physiologically relevant membrane voltage range, these inhibitors tend to block I_Na,early_ more and more, thus losing their selectivity [153]. Secondly, their blocking effect is rate-dependent, in other words, the inhibition of Na^+^ current increases with the pacing frequency, exerting thus a lesser impact on I_Na,late_ in bradycardia, when I_Na,late_ is thought to be significantly greater [154].

**Table 1.** Summary of the effects of I_Na,late_ inhibitors

Inhibitor	Effect	Species	Ref
Ranolazine	Reduces Na^+^ dependent Ca^2+^ overload	Rat, guinea pig, rabbit, canine, human	[60,66,158,164,179,183–186]
	Inhibits I_Kr_	HEK293	[163]
	Weak inhibitor of I_Ca,L_ & NCX	Canine	73,[164]
	Weak β-adrenergic agonist	Rat	[165]
	Reduces beat-to-beat variability of APD	Canine	[167]
	Reduces beat-to-beat variability of APD, dispersion of repolarization, occurrence of EADs	Guinea pig	[75]
	Suppresses dofetilide-induced TdP	Canine	[187]
	Decreases susceptibility of sustained VF	Rabbit	[188]
	Suppresses EADs and reduces TDR	Canine	[189]
GS-458967	Suppresses and prevents EADs & DADs and focal VT & VF	Rat	[157]
	Suppresses spontaneous induction of AF	Porcine	[155]
	Suppresses dofetilide-induced TdP	Canine	[190]
	Suppresses isoprenaline- and high Ca^2+^-induced DADs	Canine	[191]
	Suppresses autonomically triggered AF	Porcine	[155]
	Suppresses catecholamine-induced VT & TWA	Porcine	[192]
	Decreased susceptibility of sustained VF	Rabbit	[188]
	Decreases APD, reverse-rate dependence, triangulation, QT and TDR	In Silico	[159]
Mexiletine	Reduces TDR and prevents TdP in models of LQTS-2 & LQTS-3	Canine	[170]
	Shortens QTc, effective in treating TWA & TdP	Human	[193]
	Reduces the occurrence of polymorphic VT in simulated LQTS-2	Rabbit	[194]
	Shortens QT in LQTS-2 & LQTS-3	Human	[195–197]
F15845	Shortens APD & QT	Rabbit	[198]
	Prevents ischemia-induced VT & VF	Rabbit	[198]
	Inhibits regional myocardial ischemia-induced ST elevation	Rabbit	[199]
	Inhibits epicardial ST elevation	Canine	[199]
Eleclazine (GS-6615)	Decreases spatiotemporal dispersion of repolarization	Rabbit	
[172]			
	Suppresses catecholamine-induced VT & TWA	Porcine	
[160]			

Na^+^ channel inhibitors can differ in their selectivity, some inhibits I_Na,late_ more potently than I_Na,early_. The key issue with the I_Na,late_ blockers that they inhibit I_Na,early_ more potently at physiological membrane potentials and their rate-dependent behavior; namely, they have a lesser impact in bradycardia, where I_Na,late_ is elevated. The hallmark of I_Na,late_ inhibition is the suppression of Ca^2+^-dependent triggered activities, by reducing [Na^+^]_i_ and [Ca^2+^]_i_. The second feature of I_Na,late_ blockade is the normalization of repolarization, allowing restoring the repolarization reserve. AF, atrial fibrillation; APD, action potential duration; DAD, delayed afterdepolarization; EAD, early afterdepolarization; I_Ca,L_, L-type Ca^2+^ current; I_Kr_, rapid component of delayed rectifier K^+^ current; LQTS, long QT syndrome; NCX, Na^+^-Ca^2+^ exchange; TdP, Torsade de Pointes ventricular tachycardia; TWA, T-wave alternans; VF, ventricular fibrillation; VT, ventricular tachycardia.

GS-458967, like other Na^+^ channel blockers including ranolazine and mexiletine, reduces APD and suppresses EAD or DAD formation and generation of Torsade de Pointes (TdP) type ventricular tachyarrhythmias [18,155–159]. F15845, an anti-ischemic drug, was also shown to inhibit I_Na,late_ and prevent ventricular tachycardia and fibrillation [155]. The latest promising inhibitor compound was the eleclazine (GS-6615) which has undergone clinical trials. Eleclazine was demonstrated to bind to the Na^+^ channels with rapid kinetics and block I_Na,late_ with minimal effects on other ion currents and without adverse side effects [160]. Eleclazine shortened APD and the QT-interval, decreased spatiotemporal dispersion of repolarization, and suppressed the epinephrine induced ventricular tachycardias. Despite the encouraging results with eleclazine, the drug and the clinical trials were suspended as the number of implantable cardioverter defibrillator (ICD) shocks was higher in the eleclazine-treated group. The use of amiodarone, a blocker with mixed effects, seemed to be a promising drug in HF, but there was a higher incidence of QT prolongation and bradycardia or pulmonary fibrosis, hepatotoxicity, and thyrotoxicity [161].

The most extensively studied selective I_Na,late_ inhibitor is the antiischemic ranolazine. Ranolazine reduces Na^+^ dependent Ca^2+^ overload by inhibiting I_Na,late_ [162]. This compound also inhibits the rapid component of the delayed rectifier K^+^ current (I_Kr_) [163], I_Ca,L_ [73] and reverse mode NCX [164]; however, effects on I_Ca,L_ and NCX are mainly out of the therapeutic concentration range of the drug. Ranolazine is also a weak β-adrenergic agonist [165], while it has minimal effects on blood pressure or heart rate [166]. Furthermore, ranolazine reduced the beat-to-beat variability of APD [167]. Unfortunately, ranolazine shares the same disadvantages, namely, the enhancement of I_Na,early_ inhibition in the case of partially depolarized membrane observed in diseased hearts or at higher activation rates [168,169]. At low pacing rates inhibition of I_Na,late_ successfully decreased the arrhythmic events, such as EADs, DADs or TdP [45,53,75,170–172]. Ranolazine reduced the dispersion of repolarization [173] the occurrence of EADs and TdP [75,174]. Dispersion of repolarization is caused by the shortening of the APD of midmyocardial cells, where I_Na,late_ is the most prominent. In LQTS-3 patients ranolazine decreased [175], while in a different study it increased the QT interval, due to I_Kr_ blockade [176].

It has been demonstrated that suppression of I_Na,late_ hinders Ca^2+^ overload [167,177–179]. The hallmark of I_Na,late_ inhibition is the suppression of Ca^2+^-dependent triggered activities, by reducing [Na^+^]_i_ and [Ca^2+^]_i_. A second feature of the I_Na,late_ blockade is the normalization of repolarization, allowing restoring the repolarization reserve [55].

Characterization of the true gating mechanism of I_Na,late_ (the potential gating mechanisms were discussed previously) may also bring a better therapeutic protocol closer, since each gating mechanism has its own characteristic drug affinity and sensitivity profile [164,180,181].

## Conclusions

In summary, we have reviewed the arrhythmogenic behavior of the augmented late Na^+^ current and the concomitant elevation of intracellular Na^+^ and Ca^2+^ concentrations. In spite of the tremendous work that had been done on understanding the background of the Ca^2+^ related arrhythmias, there is still a need for more research to better design antiarrhythmic treatments and drugs. It became clear that more attention has to be paid to I_Na,late_ in patients with Ca^2+^-dependent arrhythmias, especially in the case of bradycardia.

Various processes can lead to Ca^2+^ overload and the therapeutic options are rather complex if we take into account the augmented I_Na,late_. Hence, a selective I_Na,late_ inhibitor may only treat a part of the issue. To date, the most selective drug to I_Na,late_ in the market is ranolazine; however, it exerts electrophysiological effects on other ion currents as well. Therefore, more work is necessary to gain a better understanding of the role of I_Na,late_ and Ca^2+^ handling in cardiac arrhythmias and to develop novel antiarrhythmic therapies with a focus on translational aspects.
